# A simulation of increasing rice price toward the disparity of income distribution: An evidence from Indonesia

**DOI:** 10.1016/j.heliyon.2023.e13785

**Published:** 2023-02-17

**Authors:** Fitrawaty, Wawan Hermawan, Muhammad Yusuf, Indra Maipita

**Affiliations:** a Faculty of Economics, Universitas Negeri Medan, Indonesia; b Faculty of Economics and Business, Universitas Padjadjaran, Indonesia

**Keywords:** Exchange rate, International rice price, GDP Per capita, Rice price, Disparity, Income distribution

## Abstract

**Purpose:**

Considering the significance of rice as a staple food in Indonesia, this study aims to analyze the impact of various factors, i.e., domestic price production level, exchange rate, international rice price, and Gross Domestic Product (GDP) per capita, on domestic rice prices and the resultant disparity in income distribution and inequality in the country.

**Methods:**

A simulation analysis has been performed to assess the impact of the above-mentioned factors on rice prices and the resultant income disparity in Indonesia. For that, input data from 2006 to 2020 was used to depict the change in domestic rice prices from 2021 to 2026 due to independent variables changes.

**Findings:**

Results revealed that a regular increase in rice production decreases rice prices in the longer term. Besides, a rise in the exchange rate decreases rice prices, and a fall in the exchange rate results in higher rice prices. Results also showed the insignificant impact of international rice prices on domestic rice prices in Indonesia. In contrast, an increase in per capita income reflected an increase in rice prices. Moreover, the result of the study exemplifies that the expenditure for rice has a very low elasticity (0.0975) compared to the expenditure on non-rice food (0.4096). More than half of the total household expenditure (50.71%) is spent on food, while the rest (49.29%) is used for non-food. The increase in rice prices affects the rise of income amongst farmers and declines the income of non-farmers. Hence it affects the decline of disparity in households' income distribution.

**Originality/value:**

This study adds value to the existing literature with several implications for practitioners, policymakers, and government organizations to take necessary measures to stabilize rice prices and income distribution among the various income groups.

## 1 Introduction

More than 95% of Indonesians favor rice as the staple food. Hence, rice is a prime commodity that strongly influences the poverty line [1]. Besides, the per capita rice consumption of the Indonesian nation in 2017 was calculated at 97.6 kg, whereas in 2020, it reached 140 kg [2]. Moreover, Indonesian rice consumption per capita is higher than in the neighboring nations, where rice is also considered a staple food [3]. For instance, Malaysia's per capita rice consumption was 87.9 kg, and Brunei Darussalam was recorded as 71.1 kg per capita in 2017 (Ministry of Primary Resources and Tourism, 2017). Researchers also projected that rice consumption by Indonesians is likely to increase in the future based on the growing population and increase in income level of the individuals [1]. Simultaneously, population is projected to increase from 270 million people in 2020 to 319 million people in 2045 (Khazanah Research Institute 2019). Hence, it is of utmost significance to analyze the future rice prices and their impact on income distribution and poverty level in the country as research reports inequality of income distribution among different groups in Indonesia.

For instance, in 2020, 85 million people were reported to belong to the middle-income group, which is projected to reach 223 million people in 2045 [4]. Hence, it is also projected that this inequality will rise in the future based on less rice production and higher prices. At the same time, compared to rice's growing demand in Indonesia, its production is declining. Following the US department of agriculture's data, the milled rice production in Indonesia has decreased from 38.31 million tonnes in 2008 to 33.5 million tonnes in 2019. Simultaneously an “Area Sampling Frame (Kerangka Sample Area/KSA)” method conducted by Statistics Indonesia in 2018 and 2019 reported that in 2018 total national rice production was 59.2 million tons which decreased to 54.6 million tons in 2019. This is further likely to create competition and purchasing power for various income groups. Hence, it is significant to forecast future trends in rice prices and their outcomes [1].

Moreover, over the past decades, the economic inequality issue within the country has been a topic of most interest among economic historians and economists [5,6]. Various scholars reported different reasons for this economic disparity among the various groups in a nation. Besides, this study contributes to the existing literature by examining the impact of rice prices in Indonesia on economic inequality based on income distribution. For instance, in September 2018, food commodities in Indonesia contributed the highest point on the poverty line either in rural or urban, which portion reaches 18.31% percent of the poverty line in the urban area and 25.33% of the poverty line in the rural area [7].

Simultaneously, rice prices in Indonesia are one of the economic issues long discussed in international markets based on higher prices compared to the other producer countries [8]. More than in the last decade, the price of domestic rice has been higher than international rice. Even though both recorded increases, the gap is widening [9]. Since 2010, domestic rice prices have been 60% higher than international rice prices [10]. Moreover, since 2018, rice prices in Indonesia's local markets have reached US $1.00 per kilogram, approximately twice as compared to the other neighboring countries [8]. This might be because of the long rice supply chain, the lack of advantages available to the rice farmers, and the lack of policy controls. These issues need to be addressed in the longer term to avoid the issues of income inequality and the rice crisis.

Furthermore, the high rice price can escalate poverty and disparity in income distribution [11,12]. At the same time, Asia Development Bank (ADB) research forecasted that 20% of the rise in food prices could increase the level of disparity by as many as 1% in the Gini coefficient [13]. Besides, several factors, including future expectations, price level, income level, and interest rate, influence a household's spending on consumption [14]. Likewise, based on Keynes's Consumption theory, the consumption level of a person is highly influenced by income [15]. This means when prices rise, purchasing powers decline because the money spent to maintain the same level of consumption increases [15].

At the same time, research shows that rice prices influence the poverty level, purchasing power, and disparity in the nations where rice is considered a staple food [11,12]. In connection to that, Hasan [16], in his research, found that the rise in rice prices will escalate per capita income disparity. More recently, Balié, Minot [17] reflected the positive association between rice trafficking and rice prices with poverty in the Philippines. Simultaneously, by utilizing the method applied by Badolo and Traore [18], we have done a study on the impact of rice prices on poverty and income disparity in Indonesia. From the above-stated statistics and literature support, it is paramount to say that analyzing the impact of the rise of rice prices needs to be done because it threatens the disparity of income distribution. This generally happens in low-income countries, importer countries, and some developing countries [11]. Hence, the current study aims to examine the impact of domestic price production level, exchange rate, international rice price, and GDP per capita on the rise in rice prices and the resultant income disparity in Indonesia.

Moreover, the rest of the study is organized as follows. The next section presents the research methods, followed by the results and discussion. Section four of the article concludes and provides elements for consideration with limitations and future research directions.

## 2 Data and methodology

Furthermore, simulation analysis has been performed to assess the impact of various factors, including domestic price production level, exchange rate, international rice price, and GDP per capita, on rice prices and the resultant income disparity in Indonesia. Besides, simulation analysis reflects developing the graphical and mathematical representation of future phenomena based on the existing data [19]. Demand for rice in the form of price function is assumed to be influenced by the production level of domestic rice, the exchange rate, the price of international rice, and per capita income produced by GDP per capita. In the mathematic equation, this can be translated into [1].

(1) $$\ln\;P_{{dom}_{t}}=a+\beta_{1}\;\ln\;Q_{{dom}_{t}}+\beta_{2}\;\ln\;exc_{t}+\beta_{3}\;\ln\;P_{{\mathrm{int}}_{t}}+\ln\;I_{{cap}_{t}}+u_{t}$$

where; *P*_*dom*_ = the price of domestic rice, *Q*_*dom*_ = the production of domestic rice, *Exc* = exchange rate Rupiah (RP)/USD, *P*_*int*_ = the price of international rice, *I*_*cap*_ = GDP Per capita, and *t* = the year.

The rice price behavior can be derived using the equation model (1). For that, we used the input data from 2006 to 2020 to depict the change in domestic rice prices from 2021 to 2026 due to the changes in independent variables. The coefficient (Coef) value of Eq. (1) contributes to depicting the behavior of the changes of four variables. These four variables have become the foundation in executing the simulation by changing the rising percentage of every variable. Moreover, to depict the change in domestic rice prices from 2021 to 2026, five types of simulations have been done by increasing or declining the value of each variable. These include [1] the simulation of domestic price production level [2]; the simulation of exchange rate [3]; the simulation of international rice price; and [4] the simulation of GDP per capita. The values or scores of simulations are obtained from the geometric average of growth in respectable variables (percentage) from 2006 to 2020. The scenario simulation of 1 to 4 (SIM1-SIM4) can be partially executed by doing the shock on one variable and determining that other variables are constant. Besides, scenario 5 (SIM 5) was done simultaneously by changing all independent variables. The summary of the scenario simulation is exhibited in Table 1.

**Table 1:** Scenario simulations (%).

	SIM 1	SIM 2	SIM 3	SIM 4	SIM 5
*Q*_*dom*_	±3.35				+3.35
*Exc*		±1.87			−1.87
*P*_*int*_			±5.56		+5.56
*I*_*cap*_				±3.92	+3.92

The change of price for *shock* from the four variables is the base or foundation to view the total change of expenditure in household consumption which refers to the data from Susenas (National Socioeconomic Survey). This is the way to overview the behavior of household's expenditure on rice through the weight of elasticity of rice demand and other expenditures with one condition: *constant return to scale*. To obtain the weight of household expenditure on rice and non-rice, Eq. (2) is used.

(2) $$\ln\;EXP_{i}=a+b_{1}\;\ln\;Rice_{i}+b_{2}\;\ln\;NRice_{i}+u_{t}$$

where *rice* is household expenditure on rice, *NRice* is household expenditure on non-rice, and Exp is the total household expenditure.

From the result of the change in household expenditure obtained from Susenas, the percentage of households is generated based on its expenditure. Every percentage is compared with the percentage of baseline data for respective simulations and measures the Gini index to view the disparity level in income distribution. The data used in this research is mostly secondary data consisting of Susenas and macroeconomic indicator data obtained from Indonesia's central statistical Agency (BPS), Bank of Indonesia, and other relevant and trusted sources.

## 3 Results and discussion

### 3.1 Descriptive analysis

The descriptive analysis was performed before to assess the psychometric properties of the study constructs. For that purpose, the values of skewness and kurtosis were assessed. The results revealed the values of skewness and kurtosis were within the −2 and +2 acceptable range, depicting the normal distribution of the study variables with mean values, and no potential outliers were found. Additionally, the data included in the current study did not contain any missing values. Simultaneously, correlation among the study constructs was conducted to address multicollinearity issues and establish their discriminant validity. However, results reveal that all the correlations among the study variables were less than 0.70 revealing no multicollinearity issues [20]. Simultaneously, the Variance Inflation Factor (VIF) values for study constructs were below 03, under the range of 2.1–2.4, indicating no collinearity issues [21].

### 3.2 The simulation equation of domestic rice price

Using the data from 2006 to 2020, Eq. (3) is obtained as equation estimation (1). Furthermore, this result is being used as the base to predict the impact on domestic rice prices until 2020.

(3) $$\ln\;P_{{dom}_{t}}=21.87996-0.9761\;\ln\;Q_{{dom}_{t}}-0.5092\;\ln\;exc_{t}+0.04456\;\ln\;P_{{\mathrm{int}}_{t}}+3.5985\;\ln\;I_{capt}$$

R^2^ = 0.9821; *: Significant: *exc* on alpha 10%; ***; and *I*_*cap*_ on alpha 1%.

### 3.3 The simulation on production variable of domestic rice, “q_dom_” (SIM 1)

**Fig. 1:**
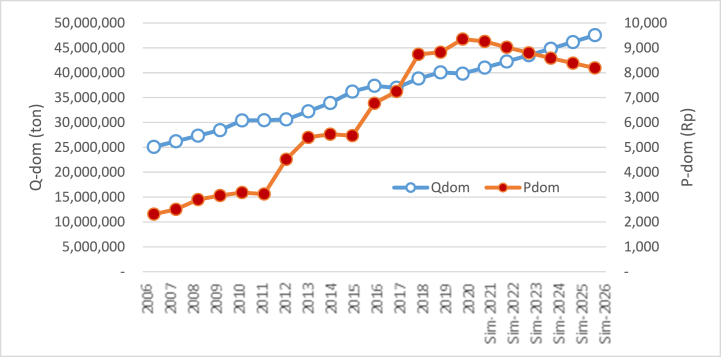
The simulation result of the rise of domestic rice production on the price of domestic rice.

The first simulation was conducted by increasing and declining domestic rice (Qdom) production level by ± 3.5% per year from the previous year. The result of the simulation is exhibited in Table 2 and Fig. 1.

**Table 2:** The result of simulation effect on the increase and decline of domestic rice production on the price of domestic rice.

			Delta/Growth				Delta/Growth	
Years	Q_dom_↑ (%)	P_dom_ (RP)	Rp	%	Q_dom_↓ (%)	P_dom_ (RP)	Rp	%
**2020**		9352.27				9352.27		
**2021**	+3.35	9257.26	(95.01)	(1.02)	−3.35	10,054.57	702.30	7.51
**2022**	+3.35	9021.99	(235.27)	(2.54)	−3.35	10,394.61	340.04	3.38
**2023**	+3.35	8798.52	(223.47)	(2.48)	−3.35	10,746.15	351.54	3.38
**2024**	+3.35	8585.97	(212.54)	(2.42)	−3.35	11,109.57	363.43	3.38
**2025**	+3.35	8383.57	(202.40)	(2.36)	−3.35	11,485.29	375.72	3.38
**2026**	+3.35	8190.60	(192.97)	(2.30)	−3.35	11,873.72	388.43	3.38
**Average**		8706.32	(193.61)	(2.18)		10,943.98	420.24	4.07
**Range**		(1161.67)				2521.45		

Where, *Q*_*dom*_ = the production of domestic rice; *P*_*dom*_ = the price of domestic rice.

Generally, the rise of domestic rice production affects the decline of domestic rice prices. The average rise of rice production of 3.35% from 2020 to 2026 has caused the decline of rice price by Rp 213,332/kg, or equal to 2.42% per year. The increase of rice production by 3.35% in 2020 has declined the domestic rice price by Rp 9257/kg in 2021. The continuity of the rise in rice production by 3.5% until 2026 continually impacts the decline of rice prices, with the percentage decline getting smaller. The rise of rice production from 2021 to 2022 would decline the rice price by 2.54% or equal to Rp 23,527/kg. But, the increase of rice production with the same percentage in 2026 declines the price by 2.30% from the previous year or equal to Rp 19,297/kg. If rice production regularly increases by 3.5% until 2026, there will be a decline in rice price by 11.52% from 2020, or equal to Rp 1,066,66/kg.

**Fig. 2:**
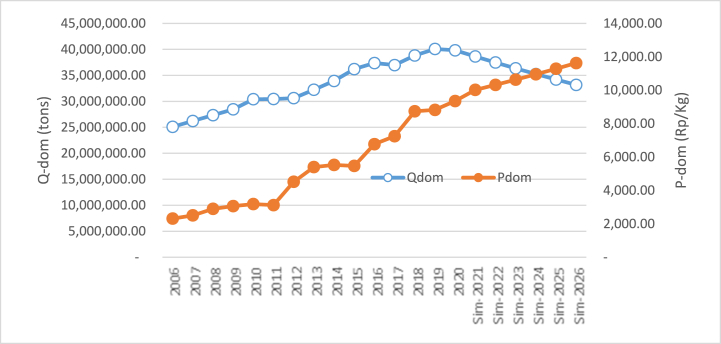
The simulation result of the decline of domestic rice production on domestic rice price.

Moreover, results also revealed that the average decline of domestic rice production per year by 3.5% until 2026 would cause an increase in the average domestic rice price by 3.38% or equal to Rp 36,383/kg. If the decline in rice price by 3.5% per year recurs regularly until 2026, the rice price will increase by 18.09% from the price of 2020 or equal to Rp 181,915/kg, so that the rice price per kg would be Rp 1,187,372 from Rp 1,005,457. Visually, the simulation result of the decline in rice production can be shown in Fig. 2.

### 3.4 The simulation on exchange rate variable “Exc” (SIM 2)

The second simulation was conducted by increasing and declining the exchange rate of the Rupiah toward the Dollar by ±1.87% per year from the previous year. The simulation result is exhibited in Table 3.

**Table 3:** Simulation result of the increase and decline of rupiah exchange rate toward the price of domestic price.

Years			Delta/Growth				Delta/Growth	
	Exc ↑ (%)	P_dom_ (Rp)	Rp	%	Exc ↓ (%)	P_dom_ (Rp)	Rp	%
	Baseline 2020: Exc = Rp 12.400/USD; P_dom_ = Rp 9352.27							
**2021**	+1.87	9634.33			−1.87	9819.59		
**2022**	+1.87	9543.86	(90.47)	(0.94)	−1.87	9914.44	94.85	0.97
**2023**	+1.87	9454.24	(89.62)	(0.94)	−1.87	10,010.21	95.76	0.97
**2024**	+1.87	9365.46	(88.78)	(0.94)	−1.87	10,106.90	96.69	0.97
**2025**	+1.87	9277.52	(87.94)	(0.94)	−1.87	10,204.52	97.62	0.97
**2026**	+1.87	9190.40	(87.12)	(0.94)	−1.87	10,303.09	98.57	0.97
Average		9410.97	(88.79)	(0.94)		10,059.79	96.70	0.97
Range		(161.87)				950.82		

**Fig. 3:**
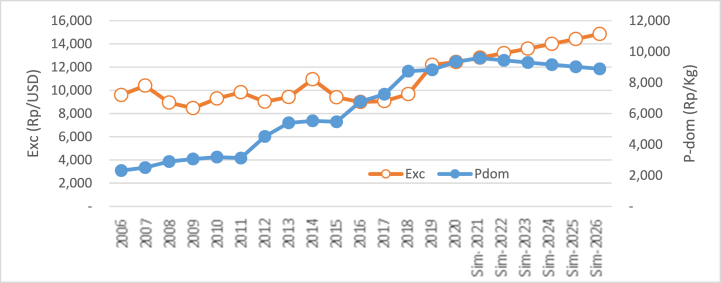
The simulation result of exchange rate (Rp/US$) on the price of domestic rice.

With an increase in the exchange rate of the local currency (the Rupiah weakens or depreciates toward the Dollar), there would be a decline in domestic rice prices. This situation follows the finding in Eq. (3). Although results show that domestic rice prices change relatively at a lower rate based on the exchange rate; however, it is statistically significant following the Eq. (3). Moreover, results reveal that when the exchange rate increases by 1.87% every year compared to the previous year, it will result in a decline in domestic rice price by 0.94% (an average of Rp 88,79/kg). Simultaneously, in 2026 the rice price is expected to decrease by Rp 443.93/kg and will reach US $ 9190.38/kg, by an increase in the exchange rate at 1.87%. Visually, the simulation result rise in the exchange rate on domestic rice price is displayed in Fig. 3.

**Fig. 4:**
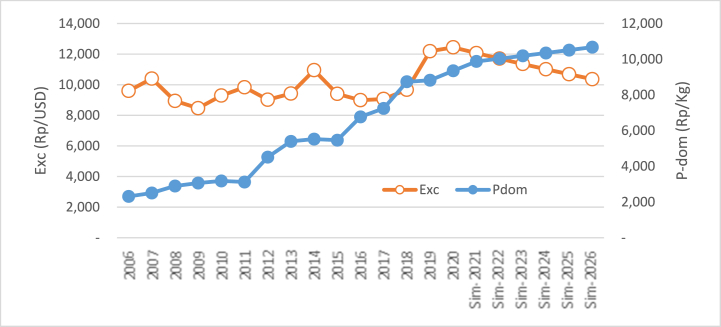
Simulation result of the decline of exchange rate (Rp/US$) on the price of domestic rice.

Conversely, a decline in the Rupiah against the dollar or rupiah appreciation triggers domestic rice prices to rise. This situation displayed in Fig. 4 shows the simulation when the decline of the exchange rate with other variables seems constant.

Moreover, from Table 3, it can be viewed that when the exchange rate decreases, the rice price increases. A regular decline of 1.87% per year in the exchange rate will result in an increase in rice prices by 0.97% per year. The little impact of the exchange rate on rice price is according to the regression result obtained in Eq. (3).

### 3.5 The simulation on the variable of international rice price “P_int_” (SIM 3)

The third simulation was conducted by increasing and decreasing international rice prices by 5.56% per year from the previous year. The result of this simulation can be viewed in Fig. 4. Generally, the increase in international rice prices impacts the increase in domestic rice prices and vice versa. Following the finding of Eq. (3), the rise of international price with the other constant variables indicates a positive coefficient of 0.05. The value of the coefficient indicates that the price of international rice has no impact on domestic rice prices.

**Fig. 5:**
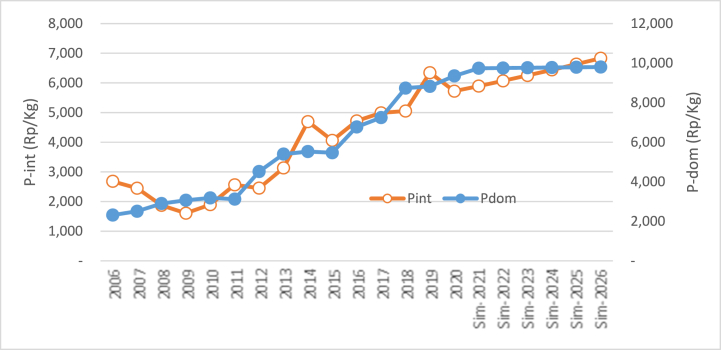
The simulation of the increase in international rice price (Rp/kg) toward the price of domestic rice.

From Table 4, it is known that within the simulation time, the average rise of international rice price is 5.56% per year, which triggers the rise of domestic rice price by 0.24% per year or equal to Rp 2365/kg per year. If the international rice price increases simultaneously by 5.56% per year from 2021 until 2026, then the rice price will increase from Rp 974,913/kg in 2021 to 986,737/kg in 2026. In other words, within five years, the rise is only Rp 118,24/kg. From Table 4, it can be concluded that the average price within the simulation is Rp. 980,816/kg. The simulation impact of the rise in international rice prices toward domestic rice prices can be viewed visually in Fig. 5. Within the simulation time, it can be seen that the pattern is horizontal, although it's not fully parallel with the horizontal axis. This indicates that the rise of international rice prices has little impact on domestic prices. The increase of international rice price by 5.56% for six years has only escalated the domestic rice price by 11,825/kg.

**Table 4:** Simulation result of the impact of the increase and decline of international rice price on domestic rice price.

Years	P_int_ ↑ (%)	P_dom_ (RP)	Delta/Growth		P_int_↓ (%)	P_dom_ (RP)	Delta/Growth	
			Rp	%			Rp	%
	Baseline 2020: P_int_ = Rp 5720.38/Kg; P_dom_ = Rp 9352.27							
**2021**	+5.56	9749.13			−5.56	9700.89		
**2022**	+5.56	9772.67	23.53	0.24	−5.56	9676.20	(24.70)	(0.25)
**2023**	+5.56	9796.26	23.59	0.24	−5.56	9651.56	(24.63)	(0.25)
**2024**	+5.56	9819.91	23.65	0.24	−5.56	9626.99	(24.57)	(0.25)
**2025**	+5.56	9843.61	23.71	0.24	−5.56	9602.48	(24.51)	(0.25)
**2026**	+5.56	9867.37	23.76	0.24	−5.56	9578.04	(24.45)	(0.25)
**Average**		9808.16	23.65	0.24		9639.36	(24.57)	(0.25)
**Range**		515.10				225.77

**Fig. 6:**
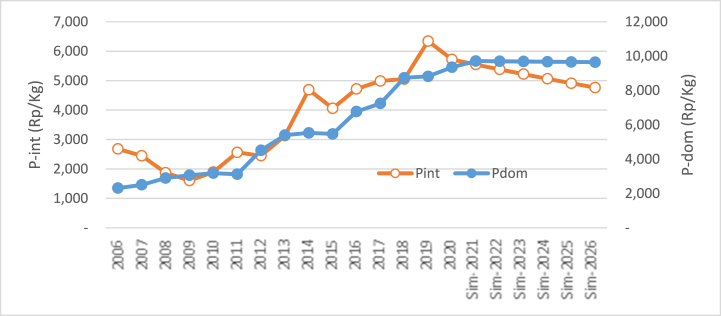
Simulation results for the decline in international rice price (Rp/kg) toward the price of domestic price.

The simulation of decreasing the price of international rice has little impact as well toward the rise of domestic rice prices. From Table 4, it can be seen that the average decline of 5.56% in international rice prices within the simulation time (2021–2026) affects the average decline of rice price by 0.254%. This number is not significant. In other words, it can be concluded that the decline of international rice prices doesn't significantly affect domestic prices. This is due to the coefficient of the change, which is relatively small, so the impact on the international price change is not obvious. In Fig. 6, it can be seen that the pattern is somehow horizontal in the simulation period, although it's not fully parallel with the horizontal axis.

### 3.6 The simulation on the variable of per capita income, “I_cap_” (SIM 4)

The rise of per capita income by 3.92% per year from the baseline (previous year) will increase domestic prices. Otherwise, the decline in per capita income decreases the price of domestic prices. This happens because per capita income and domestic rice prices have a unidirectional relationship (see Eq. (3)). The simulation result of the rise and decline in per capita income in the price of domestic prices can be seen in Table 5.

**Table 5:** Simulation result of the impact of the rise and decline in per capita income on the price of domestic rice.

Years	I_cap_ ↑ (%)	P_dom_ (RP)	Delta/Growth		I_cap_ ↓ (%)	P_dom_ (RP)	Delta/Growth	
			Rp	%			Rp	%
	Baseline 2020: I_cap_ = Rp 11.54 million; P_dom_ = Rp 9352.27							
**2021**	+3.92	11,168.92			−3.92	8422.16		
**2022**	+3.92	12,826.35	1657.44	14.84	−3.92	7293.36	(1128.79)	(13.40)
**2023**	+3.92	14,729.75	1903.40	14.84	−3.92	6315.86	(977.51)	(13.40)
**2024**	+3.92	16,915.61	2185.86	14.84	−3.92	5469.36	(846.49)	(13.40)
**2025**	+3.92	19,425.84	2510.23	14.84	−3.92	4736.32	(733.04)	(13.40)
**2026**	+3.92	22,308.58	2882.74	14.84	−3.92	4101.53	(634.79)	(13.40)
**Average**		16,229.17	2227.93	14.84		6056.43	(864.13)	(13.40)
**Range**		12,956.31				(5250.74)		

**Fig. 7:**
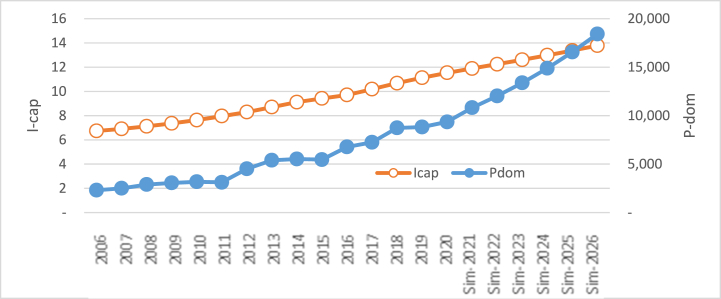
The simulation result of the rise in per capita income (million rupiah) on the price of domestic rice.

**Fig. 8:**
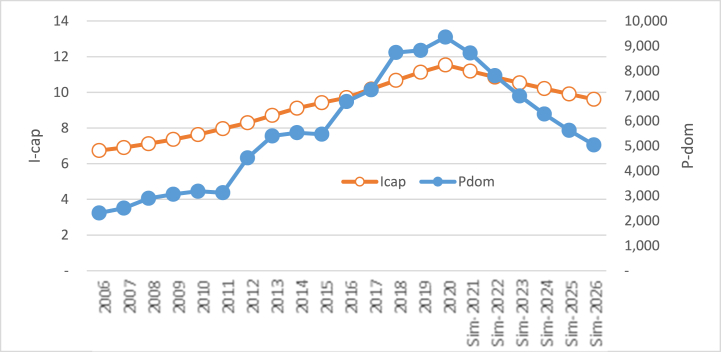
The simulation result in the decline of per capita income (million rupiah) on the price of domestic rice.

If per capita income increases by 3.92% per year from the baseline (others are constant), then domestic rice prices will increase by 14.84% every year. Otherwise, the decline in per capita income with the same amount every year will decrease domestic rice prices by 13.40% every year. The huge impact of per capita income on domestic rice prices follows the huge regression coefficient from the variable of per capita income, as shown in Eq. (3). Visually, the influence of the rise in per capita income on domestic rice prices can be seen in Fig. 7, which depicts the rise in domestic rice prices. Otherwise, in Fig. 8, the picture depicts the opposite movement. The decline in per capita income decreases the price of domestic rice.

### 3.7 The simulation on every variable simultaneously (SIM 5)

The last simulation has been conducted on four variables [1]: rice production rises at 3.35%. The assumptions are the availability of new quality seeds, advanced agricultural technology, and the provision of more paddy fields [2]; the appreciation of the Rupiah against the Dollar by 1.87%. The assumptions are better economic growth in Indonesia, better export [3]; the price of international rice increases by 5.56% due to the increasing demand for rice in the world; and [4] the increase in per capita income by 3.92% due to the good climate of the Indonesian economy. The impact of this simulation on domestic rice prices can be seen in Table 6.

**Table 6:** The simulation result of the impact of simultaneous change (the production of domestic rice, exchange rate, international price, and per capita income) on the price of domestic rice.

Year	Q_dom_↑3.35%	Exc ↓−1.87%	P_int_↑5.56%	I_cap_↑3.92%	P_dom_ (Rp)	Delta/Growth	
						Rp	%
***2020***	*39,823,915.00*	*12,440.00*	*5720.38*	*11.54*	*9352.27*		
**2021**	41,018,632.45	12,066.80	5892.00	11.88	10,687.88	1335.61	14.28
**2022**	42,249,191.42	11,704.80	6068.76	12.24	11,745.30	1057.42	9.89
**2023**	43,516,667.17	11,353.65	6250.82	12.61	12,907.34	1162.04	9.89
**2024**	44,822,167.18	11,013.04	6438.34	12.98	14,184.35	1277.01	9.89
**2025**	46,166,832.20	10,682.65	6631.49	13.37	15,587.71	1403.35	9.89
**2026**	47,551,837.16	10,362.17	6830.44	13.78	17,129.91	1542.20	9.89
**Average**	44,220,887.93	11,197.19	6351.97	12.81	13,707.08	1296.27	10.62
**Range**	7,727,922.16	(2077.83)	1110.05	2.24	7777.64	1542.20	9.89

**Fig. 9:**
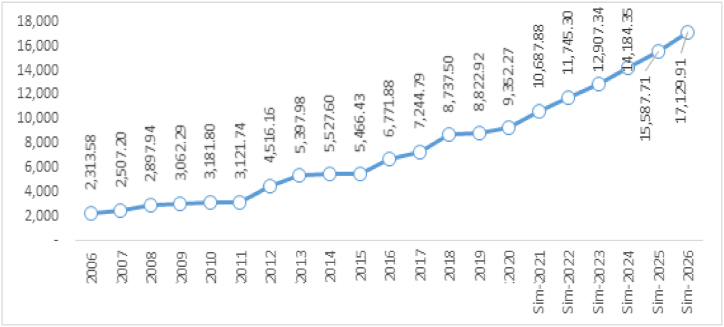
The simulation results on the change of variable of rice production, the decline of exchange rate, the rise of international rice price, and the increase of per capita income.

The impact of the simultaneous change from this scenario has triggered a rise in the average domestic rise price by 10% per year. Although the rise in domestic rice production and the decline of the exchange rate partially decreases the price of domestic rice, simultaneously, both of these variables are unable to be equal to the rising price caused by the rise in international rice price and per capita income, so that the resultant of that simulation increases the price of domestic rice. Visually, the impact of this simulation can be seen in Fig. 9, where the rise is high in domestic rice prices. The biggest contributor to this high increase is the rise in per capita income.

### 3.8 The simulation of household expenditure equation

The change of price in four variables that have been conducted before becomes the foundation to overview the change in the total household consumption expenditure, which refers to the data from Susenas. This way is to overview the behavior of household expenditure on rice through the weight or scale of elasticity of rice demand and other expenditure with one condition: a *constant return to scale* (*b*_*1*_ + *b*_*2*_ + *b*_*3*_ = 1). A conditional regression needs to be done to see the weight or scale of household expenditure on rice, non-rice, and non-food by using Eq. (4).

(4) $$\ln\;EXP_{i}=a+b_{1}\;\ln\;Rice_{i}+b_{2}\;\ln\;NRice_{i}+b_{3}\;\ln\;NFood_{i}+u_{t}$$

where *Rice* is household expenditure on rice, *NRice* household expenditure on non-rice, *Nfood* is household expenditure on non-food, Exp *is the* total household expenditure. This regression signs a condition that the total of the coefficient in expenditure for rice (*Rice*), expenditure of non-rice (*NRice*), and expenditure of non-food (*NFood*) must be equal with one (*constant return to scale*). The data used is Susenas of the consumption model for 2018–2021. The summary of the regression result for respective years can be seen in Table 7. At the same time, the complete result of regression for each year can be seen in the appendices.

**Table 7:** The regression result of conditioned household expenditure.

Variable	2018	P > t	2019	P > t	2020	P > t	2021	P > t	Average
lrice	0.1024	0	0.1000	0	0.0909	0	0.0966	0	0.0975
lnRice	0.4078	0	0.4081	0	0.4191	0	0.4037	0	0.4096
lnfood	0.4898	0	0.4920	0	0.4901	0	0.4997	0	0.4929
_cons	1.0237	0	1.0206	0	1.0040	0	1.0103	0	1.0146

Source: Susenas data 2018–2021; processed.

The regression result shows that all independent variables are significant at the level of 1% alpha for every year. The coefficient average (elasticity) from the conditioned regression variable is displayed in the rightmost column of Table 7. Using the average score, Eq. (4) can be rewritten into Eq. (5).

(5) $$\ln\;EXP_{i}=1.013+0.0975\;\ln\;Rice_{i}+0.4096\;\ln\;NRice_{i}+0.4929\;\ln\;NFood_{i}$$

Equation (5) signifies that expenditure for rice has very low elasticity, that is, 0.0975%. It means if there is spending on rice by 1%, it will increase the household expenditure by 0.0975%. The expenditure for non-rice food shows more elasticity toward the total household expenditure. The elasticity is 0.4096%. This means if there is expenditure for non-rice food by 1%, it will increase the household expenditure by 0.4096%. The household expenditure for non-food has the biggest portion of the total expenditure, that is 0,4929%. This means if there is an increase in the expenditure on non-food by 1%, it will increase the total household expenditure by 0.4929%.

Moreover, the phenomena in the conditioned regression equation depict that expenditure on rice is the smallest portion of spending from the total household expenditure. The expenditures for non-rice food and non-food have almost the same portion, that is, 0.4096% and 0.4929, respectively. In total, the expenditure on non-food is slightly smaller than on food. This means almost half of the expenditure (50.71%) is spent on food, and the rest (49.29%) is spent on non-food. Furthermore, from Eq. (5), it can also be concluded that within 2018–2021, it's only 10% of the total household expenditure spent on rice. From the total household expenditure for food (50.71% of the total expenditure), it's only 19.23% spent on rice, while the rest of 80.77% is spent on non-rice food. This situation shows the behavior of household expenditure in Indonesian society to fulfill their necessity for food. Completely, rice demand elasticity on income produced from the total expenditure within 2018–2021 is displayed in Table 8.

**Table 8:** Rice Demand Elasticity (kg) on Income, The year 2018–2021.

Year	Elasticity
2018	0.18
2019	0.18
2020	0.17
2021	0.18

Source: Susenas Data 2018–2021; processed.

Table 8 depicts that rice demand elasticity on income level is relatively alike within the research year, that is 0.18%. This means if the income increases by 1%, it will impact the rice demand by 0.18%. This small increase shows that rice consumption is high before the income increases. According to Marktanner and Noiset [22], the level of per capita income has a reverse relationship with the expenditure on food; hence the absolute point or score of elasticity in food price toward real income level is also comparatively reversed with the expenditure on food. In other words, the bigger the portion of spending on food, the more real income experiences the decline due to the rise of food prices.

To get the simulation's impact on domestic rice price changes, the average change of geometric percentage is utilized from the domestic rice price from 2006 until 2020. The calculation result obtains that the average change of geometric percentage is 10.74%. This number mirrors the huge change in domestic price due to the change of variables that simultaneously influence the domestic rice price (variable in Eq. (3)). The score of geometric average change is being simulated on the rice price obtained from Susenas data in 2018–2018 and is estimated using Eq. (6). This equation is a *bridging equation* that is the equation of rice demand on rice price that eventually will be substituted into Eq. (5).

(6) $$Q_{rice}=a+bP_{rice}+e$$

where: *Q* is the total rice demand (kg) and *P* is the rice price (Rp).

By changing the demand *Q* and price *P* informing the natural logarithm, then from the estimation result of Eq. (6), the score or value of the rice demand coefficient based on Susenas data from 2018 to 2021 is obtained. This is displayed in Table 9.

**Table 9:** The score/value of coefficient function on rice demand from 2018 to 2021.

	2018	2019	2020	2021	Average
lnP_rice_	−0.33446	−0.31106	−0.35666	−0.31312	−0.32883
_const	4.735835	4.528965	4.93998	4.564963	4.69244

Source: Susenas Data 2018–2021; processed.

Furthermore, Eq. (6) can be rewritten as Eq. (7), that is:

(7) $$\ln\;Q_{rice}=4.6924-0.3288\;\ln\;P_{rice}$$

To obtain the equation of new total expenditure of households, Eq. (7) is being substituted into Eq. (5), as shown in Eq. (8).

(8) $$\ln\;EXP_{i}=1.013+0.0975\left(4.6924-0.3288\;\ln\;P\right)+0.4096\;\ln\;NRice_{i}+0.4929\;\ln\;NFood_{i}$$

where ln*Q* = ln*Rice*.

From the regression result of conditioned Eq. (8), it is known that the composition of people's expenditure for rice has changed and increased if it is compared with the regression result in Table 7. Table 10 displays the composition change in expenditure summary after the simulation of Eq. (8), and complete annual results are attached.

**Table 10:** Conditioned regression result after the rise of rice price by 10,47% in 2018–2021.

Variable	2018	P > t	2019	P > t	2020	P > t	2021	P > t	Average
lfood_rice	0.1124	0	0.1101	0	0.1005	0	0.1110	0	0.1085
lfood_nrice	0.3972	0	0.3985	0	0.4091	0	0.3905	0	0.3988
In food	0.4903	0	0.4914	0	0.4904	0	0.4986	0	0.4927
_cons	1.0363	0	1.0340	0	1.0173	0	1.0319	0	1.0299

Source: Calculation Result.

Based on the simulation above, there are two household expenditure data. The first is initial expenditure as the baseline, and the second is the data of expenditure which is the result of Eq. (8) as the consequence of the rise in domestic rice price. To view the impact of the rise in domestic rice price on income disparity level, Gini Index was used by comparing the Gini index before the baseline and after the simulation of the rise of rice price by 10.74%. The result of this simulation can be seen in Table 11.

**Table 11:** The impact of the rise of domestic rice price on income disparity level.

	2018			2019			2020			2021		
	Before	After	Delta	Before	After	Delta	Before	After	Delta	Before	After	Delta
GE (-1)	0.359	0.33209	−0.02691	0.34726	0.32337	−0.02389	0.3401	0.31823	−0.02187	0.38477	0.35307	−0.0317
GE (0)	0.29972	0.29286	−0.00686	0.29063	0.28465	−0.00598	0.28552	0.28026	−0.00526	0.30698	0.29975	−0.00723
GE (1)	0.36186	0.36175	−0.00011	0.34682	0.34703	0.00021	0.33877	0.33917	0.0004	0.35729	0.35714	−0.00015
GE (2)	0.83452	0.84773	0.01321	0.7869	0.79925	0.01235	0.68667	0.69613	0.00946	0.6882	0.69689	0.00869
**Gini**	**0.42112**	**0.41957**	**−0.00155**	**0.41511**	**0.4139**	**−0.00121**	**0.4115**	**0.4106**	**−0.0009**	**0.42362**	**0.42235**	**−0.00127**

Source: Susenas Data 2018–2021, the simulation result and the calculation; Before: baseline; After the simulation.

Table 11 shows that the Gini index experiences a decline before and after the simulation for every research year. This means there is a decline in income disparity levels in society. In other words, the rise of domestic rice prices by 10.74% affects the decline of income disparity levels in society (households). On the other sides, the rise in rice prices contributes to the rise of income amongst rice farmers (or at least it doesn't make them spend more). But on the customer's side, the rise of rice price increases the expenditures and declines the real income (rice is a staple food and the demand elasticity toward price is relatively smile). Because both move in the same direction, the disparity gap is getting smaller. That is why the rise of domestic rice prices impacts the decline of disparity besides the low level of elasticity of rice demand and the rise in expenditure due to the increase in the price.

Moreover, these findings are in accordance with Kuhla et al. (2021), who reported that the rise of food prices for the people's welfare relies on the combination of the decline of purchasing power and the rise of income (income effect). In households with income associated with agriculture, the rise in food prices will increase their income [23]. At the same time, it oppositely happens for those households whose income is not coming from the agricultural sector. Simultaneously, some studies by Amare, Abay [24] and Nyiwul [25] stated that the change in food price might influence the disparity through income. Likewise, Amolegbe, Upton [26] asserted that the rise in food prices decreases the disparity between the poor and the food producer.

## 4 Conclusions, implications, and future research directions

The simulation results revealed that if rice production regularly increases by 3.5% until 2026, there will be a decline of 11.52% from 2020, or equal to Rp 1,066,66/kg. Moreover, results also revealed that the average decline of domestic rice production per year by 3.5% until 2026 would cause an increase in the average domestic rice price by 3.38% or equal to Rp 36,383/kg. At the same time, the exchange rise at 1.87% every year until 2026 will result in a decrease in domestic rice price in 2026 to US $ 9190.38/kg. Likewise, if the exchange rate decline recurs regularly by 1.87% per year, then the rice price will increase by 0.97% per year. Moreover, results showed the insignificant impact of the rise of international rice prices on domestic prices. For instance, the increase of international rice price by 5.56% over six years has only escalated the domestic rice price by 11,825/Kg. In other words, it can be concluded that the decline in international rice prices doesn't significantly affect domestic prices. Moreover, if per capita income increases by 3.92% per year from the baseline (others are constant), then domestic rice prices will increase by 14.84% every year. Otherwise, the decline in per capita income with the same amount every year will decrease domestic rice prices by 13.40%. It further shows that when the purchasing power of the individual increases, rice prices also increase.

In addition, from the regression result, simulations, and discussion conducted in previous parts, it can be concluded that: (a) obtained from the regression result of household expenditure, it is known that the expenditure for rice has very low elasticity (0.0975%). At the same time, the expenditure for non-rice food has a high elasticity (0.4096%). In other words, if the expenditure for rice increases by 1%, then the total household expenditure will increase by 0.0975%; (b) more than (50.71%) half of the total household expenditure is spent on food, while the rest (49.29%) is spent for non-food; (c) within the years of 2018–2021, there is only 10% of household expenditure spent on rice. From the total household expenditure for food (50.71% of the total expenditure), it's only 19.23% spent on rice, while the rest of 80.77% is spent on non-rice food. This situation shows the behavior of household expenditure in Indonesian society to fulfill their necessity for food; (d) the rise of rice price by 10.74% impacts the decline of household income disparity level.

The study adds value to the existing literature based on the results that highlight the impact of the rise in rice prices in escalating the income disparity, particularly in urban areas or in the area where economic capability is advanced but deescalated in impoverished areas. This will further help practitioners, policymakers, and government organizations to take necessary measures to stabilize rice prices and income distribution among the various income groups. Moreover, based on the significant impact of rice production, on the rise in domestic rice prices, governments should devise specific policies defining the strict criteria of minimum and maximum production limits. Likewise, the significant impact of the exchange rate on the rise in rice prices calls for measures at the governmental level to stabilize the currency rates based on economic growth and stability so that the people with the lower-income level should not suffer based on the rise in rice prices that results due to the decrease in the exchange rate. Also, the government should take measures to stabilize the per capita income of the individuals to keep the rice prices stable and affordable for individuals belonging to different income levels in the country. It will further help bridge the gap among communities due to unequal wealth distribution. Finally, the current study has only focused on determining the influence of various factors on the change in rice prices and the resultant economic effects. However, future researchers should consider other foods like seafood and wheat to conduct the comparative analysis in terms of changes in prices on consumption patterns of the individuals and resultant economic development.

## Author contribution statement

Fitrawaty: Contributed analysis tools or data; Conceived and designed the analysis; Wrote the paper.
Wawan Hermawan: Conceived and designed the analysis; Analyzed and interpreted the data.
Muhammad Yusuf: Analyzed and interpreted the data; Contributed analysis tools or data.
Indra Maipita: Contributed analysis tools or data; Wrote the paper.

## Funding statement

Dr Fitrawaty Fitrawaty was supported by 10.13039/501100019714UNIMED [011-UNIMED-01-2021].

## Data availability statement

Data will be made available on request.

## Declaration of interest's statement

The authors declare no competing interests.

## Figures and Tables

**Table:** 

lfood_rice + lfood_nrice + lnfood = 1 (Number of obs = 60646518; Root MSE = 0.0867)						
lexp	Coef.	Std. Err.	t	P > t	[95% Conf.	Interval]
lfood_rice	0.102432	0.0000165	6225.73	0	0.1023998	0.1024643
lfood_nrice	0.4077701	0.0000245	1.70E+04	0	0.4077221	0.4078181
Lnfood	0.4897979	0.0000181	2.70E+04	0	0.4897625	0.4898333
_cons	1.023656	0.0000273	3.70E+04	0	1.023602	1.023709

**Table:** 

lfood_rice + lfood_nrice + lnfood = 1 (Number of obs = 62641051; Root MSE = 0.0871)						
lexp	Coef.	Std. Err.	t	P > t	[95% Conf.	Interval]
lfood_rice	0.0999892	0.0000162	6159.35	0	0.0999574	0.1000211
lfood_nrice	0.4080588	0.0000242	1.70E+04	0	0.4080113	0.4081062
Lnfood	0.491952	0.0000178	2.80E+04	0	0.4919171	0.4919868
_cons	1.020571	0.0000283	3.60E+04	0	1.020516	1.020627

Where: Coef = Coefficient value, and Std. Err = Standard Error.

**Table:** 

lfood_rice + lfood_nrice + lnfood = 1 (Number of obs = 63216137; Root MSE = 0.0825)						
lexp	Coef.	Std. Err.	t	P > t	[95% Conf.	Interval]
lfood_rice	0.0908521	0.0000156	5841.75	0	0.0908216	0.0908826
lfood_n rice	0.4190539	0.0000233	1.80E+04	0	0.4190083	0.4190996
lnfood	0.490094	0.0000168	2.90E+04	0	0.4900611	0.4901269
_cons	1.004032	0.0000279	3.60E+04	0	1.003977	1.004086

**Table:** 

lfood_rice + lfood_nrice + lnfood = 1 (Number of obs = 65,259,530; Root MSE = 0.0786)						
lexp	Coef.	Std. Err.	t	P > t	[95% Conf.	Interval]
lfood_ rice	0.0965667	0.0000145	6680.83	0	0.0965383	0.096595
lfood_n rice	0.4037061	0.0000217	1.90E+04	0	0.4036635	0.4037486
lnfood	0.4997273	0.0000157	3.20E+04	0	0.4996964	0.4997581
_cons	1.010291	0.0000249	4.10E+04	0	1.010242	1.010339

**Table:** 

lfood_rice1 + lfood_nrice + lnfood = 1 (Number of obs = 60646518; Root MSE = 0.0859)						
Iexp1	Coef.	Std. Err.	t	P > t	[95% Conf.	Interval]
lfood_ rice1	0,1124416	0,0000177	6354,94	0	0,112407	0,1124763
lfood_n rice	0,3972125	0,0000248	1,60E+04	0	0,3971639	0,3972611
lnfood	0,4903459	0,0000179	2,70E+04	0	0,4903108	0,4903809
_cons	1,036258	0,0000295	3,50E+04	0	1,036201	1,036316

**Table:** 

lfood_rice1 + lfood_nrice + lnfood = 1 (Number of obs = 62641051; Root MSE = 0.0862)						
Iexp1	Coef.	Std. Err.	t	P > t	[95% Conf.	Interval]
lfood_ rice1	0,1100944	0,0000175	6289,53	0	0,11006	0,1101287
lfood_n rice	0,3985232	0,0000244	1,60E+04	0	0,3984755	0,3985709
lnfood	0,4913824	0,0000176	2,80E+04	0	0,4913479	0,491417
_cons	1,033952	0,0000305	3,40E+04	0	1,033892	1,034011

**Table:** 

lfood_rice1 + lfood_nrice + lnfood = 1 (Number of obs = 63216137; Root MSE = 0.0820)						
Iexp1	Coef.	Std. Err.	t	P > t	[95% Conf.	Interval]
lfood_ rice1	0,1005407	0,000017	5929,86	0	0,1005075	0,100574
lfood_n rice	0,4090551	0,0000237	1,70E+04	0	0,4090087	0,4091016
lnfood	0,4904041	0,0000167	2,90E+04	0	0,4903715	0,4904368
_cons	1,017289	0,0000305	3,30E+04	0	1,017229	1,017348

**Table:** 

lfood_rice1 + lfood_nrice + lnfood = 1 (Number of obs = 65,259,530; Root MSE = 0.0791)						
Lexp1	Coef.	Std. Err.	t	P > t	[95% Conf.	Interval]
lfood_ rice1	0,1109593	0,0000151	7333,44	0	0,1109296	0,110989
lfood_n rice	0,3904824	0,0000213	1,80E+04	0	0,3904407	0,3905242
lnfood	0,4985583	0,0000158	3,10E+04	0	0,4985272	0,4985893
_cons	1,031923	0,0000263	3,90E+04	0	1,031871	1,031974

## APPENDIX A

Regression Result of the Conditioned Household Expenditure by Using Susenas Data from 2018 to 2021 (before simulation).

Conditioned Regression Result of 2018.

Condition Regression Result of 2019

Condition Regression Result of 2020

Condition Regression Result of 2021

## APPENDIX B

Conditioned Regression Result of the Equation of Total Expenditure After the Rise in Domestic Rice Price by 10.47% Within the Years of 2018–2021 (after simulation)

$$\ln\;EXP_{i}=1.013+0.097\left(4.7469-0.3348\;\ln\;P\right)+0.410\;\ln\;NRice_{i}+0.493\;\ln\;NFood_{i}$$

2018

2019

2020

2021
